# The Impact of Rural Livelihood Diversification on Household Poverty: Evidence from Jimma Zone, Oromia National Regional State, Southwest Ethiopia

**DOI:** 10.1155/2021/3894610

**Published:** 2021-12-23

**Authors:** Tsehaynesh Abebe, Tamiru Chalchisa, Adugna Eneyew

**Affiliations:** ^1^Gambella Agriculture Research Institution, Department of Socio-Economic and Agricultural Extension, Gambela, Ethiopia; ^2^Jimma University, Department of Rural Development and Agricultural Extension, Jimma, Ethiopia

## Abstract

In Ethiopia, agriculture is the principal source of food and livelihood for many rural households, making it a central component of programs that seek to reduce poverty and achieve food security. Since the sector is faced with many challenges, rural households are compelled to develop strategies through diversification to cope with the increasing vulnerability associated with agricultural production. As a result, the purpose of this research is to assess the impact of livelihood diversification on household poverty in the Jimma zone of Ethiopia's Oromia regional state. A multistage sampling procedure was employed to select 385 sample household heads. The study utilized data obtained from a cross-sectional survey using an interview schedule, focus group discussion, key informant interview, and personal observations. Both descriptive and econometric data analysis techniques were applied. The result of the FGT poverty measure revealed that the incidence of poverty among rural households was 37.14%, implying that 62.86% were non-poor. The descriptive statistics revealed that age of household, dependency ratio, year of schooling, sex of household, livestock ownership, landholding, non-farm income, market distance, and extension contact were found to have a significant influence on the poverty status of a household at different probability levels. Based on the cost of basic needs approach, it was applied to measure poverty status. The results of the logit model indicate that family size, landholding, livestock ownership, year of schooling, access to credit services, and off-farm income of the households were found to have significantly determined livelihood diversification. Moreover, the results of the propensity score matching indicate that household participation in livelihood diversification has a positive and significant impact on household poverty. Accordingly, households with diversified livelihoods were found to be 9% better off than those that were not diversified in terms of poverty. Policies aimed at increasing the income generation ability of the household should be strongly considered. Therefore, to ensure the capacity of rural households to practice farming along with a wide range of income-generating activities to improve the well-being of the rural poor and have a significant impact on poverty reduction, participating in livelihood diversification should be given emphasis in development planning.

## 1. Introduction

### 1.1. Background and Justification of the Study

The agricultural sector is the growth, development, and pathway to ensuring food security in sub-Saharan Africa. It is the largest employer of labor, and most poor people depend heavily on it for their livelihood as it is the only way out of poverty and food security [1]. However, subsistence producers and small-farm wage laborers in the rural areas of low-income countries constitute over two-thirds of the global poor and food insecure populations, and about 70% of the world's very poor people live in rural areas [2–4]. However, stagnant agricultural productivity and low returns from farming have led rural people to look for alternatives to supplement their income.

According to recent studies, the contribution of livelihood activities and migration to increasing rural household income accounts for 50% of rural household income in the developing world [5, 6]. On the contrary, many factors influence rural households' ability to diversify their livelihood strategies away from crop and livestock production and into off- and non-farm economic activities. According to FAO (2012), population growth is outstripping the current productive high-carry capacity of the land. As a result, 47.9% of the population in rural Africa lives in extreme poverty [8]. Therefore, diversification is highly significant for poor rural households and supports the accumulation of income for farm expansion and engagement in non-farm businesses [9, 10].

The high level of poverty in developing countries' rural communities has pushed many households into diverse portfolios in order to cope with the risks and shocks associated with agricultural production, avoid producing below the subsistence threshold, and improve their quality of life. According to Babatunde and Qaim [11], Bezu et al. [12], and Hoang et al. [13], diversification to non-farm livelihood strategies instead of depending on subsistence farming alone allows families to improve financial status, increase production, and cope with environmental stress and shock. Pingali and Rosegrant [14] also posited that livelihood diversification is an essential strategy employed to move from subsistence and poverty to commercial and prosperity, respectively.

In Ethiopia, poverty is highly correlated with the size and composition of households, the educational level of household heads, the degree and extent of dependency within the household, asset ownership (particularly ownership of oxen in rural areas), the occupation of household heads, rapid population growth, major health problems, lack of infrastructure, and extreme environmental degradation [15]. Then, maintenance of a diversified resource base is a prerequisite for adaptation to climate variability, as diversified livelihood systems allow indigenous farming communities to draw on various sources of food and income [16].

Recent evidence indicates that in Ethiopia, nearly 55% of all smallholder farmers operate on 1 hectare or less [17]. Due to the smaller farm size and low return from farming activities, the majority of rural households are engaged in diversified income sources. Similarly, Ethiopia is among the low-income countries in the world with a GDP per capita of $1,608 in purchasing power parity (PPP) terms in 2017 and ranked 164 out of 187 countries [18]. In poor countries, the new line equals $1.90 per person per day [19]. Then, the absence of off-/non-farm income opportunities leads to asset depletion and increasing levels of poverty at the household level.

Additionally, in rural areas, the agricultural sector alone cannot be relied upon as the core activity for rural households as a means of improving lives and reducing poverty. For instance, Bogale and Shimelis [20] clearly state that because of primary dependence on subsistence crop production, harvest failure leads to household food deficits in Ethiopia. Yenesew et al. [21] point out that livelihood diversification is believed to be a solution and an effective strategy for the reduction of poverty and food insecurity in rural Ethiopia. Additionally, Sisay [22] stated that off-farming activities have an impact on the level of poverty and income inequality, and where the poor have equal access to participate in high-earning off-farm activities, their impact on poverty reduction and income equality will be significant.

The propensity for rural households to engage in multiple occupations is often noted, but few attempts have been made to link this behavior in a systematic way to rural poverty reduction [23]. Like in the other regional economies of Ethiopia, households in the study area mainly depend on small-scale subsistence agriculture to derive their livelihoods. Moreover, though with the existing limited farm size, agriculture could not enable them to generate enough income to fulfill household needs; this suggests the necessity for non-farm diversification in the study area.

Furthermore, while the poverty situation is substantial, resources are available, necessitating thorough knowledge. There have been no comprehensive researches on livelihood diversification and its relationship to poverty in the zone and district. Furthermore, no research has been undertaken in this area to determine the influence of livelihood diversification on household poverty. As a result, this research aims to add to the body of knowledge by empirically linking poverty status to livelihood diversification in rural households in the Jimma zone.

## 2. Methodology

### 2.1. Description of the Study Area

**Figure 1:**
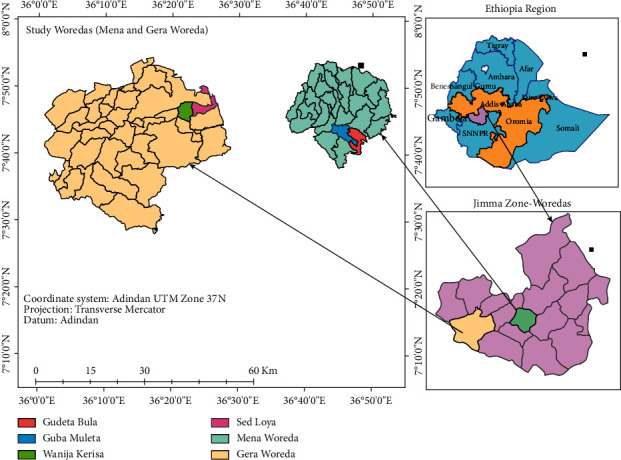
Map of the study area Ethio GIS (2019).

Based on Figure 1, the Jimma zone is one of the 18 zones of Oromia regional state located in the southwest of Ethiopia, at a distance of 352 km from the capital city of the country, Addis Ababa (Ethio GIS 2019). The zone has 21 woredas and 1 city town with a total population of 2,780,549 living in 543 kebeles [24]. The area receives annual rainfall in the range of 1,200–2,800 mm and temperatures of 16–20°C in normal years. The rainy season extends from February to November and is suitable for growing coffee, cereals, and pulses, as well as root and fruit crops. The highlands and the swampy areas grow maize and barley as early-season crops, using residual moisture in the depressions. Only 25% of farmers in the area possess one or more oxen. Despite considerable deforestation in recent years, 27% of the total area of the Jimma Zone remains forested (natural, artificial, shrubs, and bushes) [25].

### 2.2. Research Design

A cross-sectional research design was used for this research. In order to conduct the study in a representative way and to increase its reliability and validity, both purposive and simple random sampling procedures were employed.

### 2.3. Sampling Procedure and Techniques

A multistage sampling procedure was employed to select the representative households for this study. Accordingly, in the first stage, 2 woredas, namely, Gera and Mana, were purposively selected. The use of a purposive sampling technique is justified based on the engagement of the households in the diversified rural livelihood activities. Because there is no reliable data on the sample frame of diversifying households in the study area, hence, four representative samples of kebeles were selected randomly from a total of 55 rural kebeles found in both woredas (2 kebeles from each woreda). Finally, a total of 385 sample households were selected using simple random sampling techniques. A proportional population sample size was used to redistribute sample households across kebeles. The sample size of this study was determined by using the maximum sample size formula adjusted for the total population of the study area by using Cochran's sample size formula employing [26] as follows:

(1) $$\begin{array}{c} n_{o}=\frac{z^{2}*pq}{d^{2}}. \end{array}$$

### 2.4. Sources and Methods of Data Collection

Both the qualitative and quantitative methods of data collection are employed to address the basic objectives of this study. A well-structured questionnaire was used to collect primary data from 385 randomly selected sample household respondents. The data collected included the socioeconomic characteristics of the household such as the age of the household, educational status of the respondents, household size, number of livelihood activities engaged in by the household head, livestock holding, distance from the local market, land size, access to credit, number of extension contacts, off- and non-farm income, food and non-food expenditure for determining the poverty status of the household. In addition, focus group discussions, key informant interviews, and personal observations were employed in order to explore and fully describe the existing livelihood diversification strategies in the study area. Moreover, prior to the actual survey, the questionnaires were pretested on non-sample respondents for consistency and clarity and to check their validity and reliability as well as to obtain the incorporation of modifications intended for the data.

### 2.5. Methods of Data Analysis

Based on the nature of the specific objective of the study, descriptive statistics and econometric models were employed to analyze the data. Specifically, analysis of data collected from the field was done by using the cost of basic need approach (consumption expenditure), poverty status identified as nonpoor from poor households, and the logit model. To analyze the determinants of rural households' participation in livelihood diversification strategies, the propensity score matching (PSM) technique was used to generate estimates for paired treatment (diversified sample household) and control (nondiversified sample household) groups based on the similarity of observable characteristics.

#### 2.5.1. Poverty Indices Measurement

To estimate the poverty line, the study used the cost of basic need (CBN) method. To this end, a basket of food items consumed by the households were recorded and converted to calories using the food composition table developed by Ethiopia Health and Nutrition Research Institute (EHNRI 2008). The aggregate food calories were adjusted in adult equivalent units, and all that was consumed was multiplied by the local price of acquiring them to estimate the food poverty line. To account for an allowance for non-food basic needs, the non-food poverty line was determined using a simple linear regression of the share of food expenditure to total expenditure (*S*) to compute the total poverty line [28] as follows:

(2) $$\begin{array}{c} S_{i}=\alpha +\beta \;\log\frac{\left(\text{TE}\right)}{\left(\text{FPL}\right)i}+\varepsilon i, \end{array}$$

where *i* runs through the sample households 1 to *n*. After constructing the poverty line using expenses for food and non-food basic needs, the FGT poverty model (Foster–Greer–Thorbecke model) identified three poverty measures following the procedures developed by Foster et al. [29], namely, the incidence of poverty (*P*_0_), the depth of poverty (*P*_1_), and the severity of poverty (*P*_2_) are used. The FGT index is formulated as follows.

Three conditions about poverty depending upon the weight attached to *α*:

(3) $$\begin{array}{c} P\left(a\right)=\left(\frac{1}{n}\right)\sum_{i=1}^{q}\left[\frac{{\left(Z-y_{i}\right)}^{2}}{Z}\right],\alpha =0,1,2, \end{array}$$

where *P* (*a*) is the poverty measure, *Z* is the poverty line, *y*_*i*_ is the consumption expenditure level, *N* is the number of sample households, *n* is the number of poor households, and *a* is the weight given to the severity of poverty (a measure of the sensitivity of the index to poverty).

#### 2.5.2. Logit Model Specification

The logit model was applied in this study to assist in estimating the probability of household participation in livelihood diversification activities that can take one of the two values, participated or non-participated household. According to Gujarati (2004), the functional form of the logit model is presented as follows:

(4) $$\begin{array}{c} p_{i}=E\left(\frac{Yi}{Yi}\right)=\frac{1}{1+e^{-\left(\beta 0+\beta 1x1\right)}}, \end{array}$$

where *P*_*i*_ is a probability that an *i*-th household participated in livelihood diversification and ranges from 0 to 1 and *Zi* is a functional form of *m* explanatory variables (*X*) that is expressed as follows:

(5) $$\begin{array}{c} Zi=\beta +\sum \beta iXi,\;i=1,2,3,\ldots ,m, \end{array}$$

where 0 is the intercept and *i* is the slope parameters in the model. The slope tells how the log odds in favor of a given household participating in livelihood diversification change as independent variables change. If *P_i_* is the probability of a household diversifying, then 1 − *P_i_* indicates the probability that a given household did not participate in any livelihood diversification.

#### 2.5.3. Propensity Score Matching (PSM) Impact Estimation

The standard framework in evaluation analysis is to formalize the potential outcome approach ([30]; Rubin 1974). The advice on which functional form to use is available, and the discussion in favor of logit or probit models (compared to linear probability models) stems from the well-known shortcomings of the linear probability model [31]. The main pillars of this model are individuals, treatment, and potential outcomes. In the case of double treatment, the treatment indicator *D*_*i*_ equals 1 if an individual *i* receives treatment, and 0 otherwise. If diversification was by chance assigned to a household, one could judge the impact of its livelihood diversification on households' poverty by comparing the average consumption cost of basic needs, identifying the non-poor from the poor based on diversification and non-diversification. In such a case, the average treatment effect can be computed as follows:

(6) $$\begin{array}{c} Ti=Yi\left(\begin{array}{cc} D_{i} & 1 \end{array}\right);\\ Yi\left(D_{i}=0\right), \end{array}$$

where *Ti* is the treatment effect (effect due to diversification), *Yi* is the outcome on household *i*, and *D*_*i*_ is whether the household *i* has got the treatment or not (i.e., whether a household diversifies or not).

However, one should note that *Yi* (*D*_*i*_ = 1) and *Yi* (*D*_*i*_ = 0) cannot be observed for the same household at the same time. Depending on the position of the household in the treatment diversified, either *Yi* (*D*_*i*_ = 1) or *Yi* (*D*_*i*_ = 0) is the unobserved outcome (called counterfactual outcome). The variables that received the most attention in evaluation literature is the “average treatment effect on the treated” (ATT), which is defined as follows:

(7) $$\begin{array}{c} \text{ATT}=E\left(Tj\;D=1\right)\\ =E\left[Y\left(1\right)j\;D=1\right]iE\left[Y\left(0\right)j\;D=1\right]. \end{array}$$

The difference between the left-hand side of equation (7) and ATT is the so-called self-selection bias. The true parameter ATT is only identified, if

(8) $$\begin{array}{c} \text{ATT}=E\left(Y1|D=1\right)-E\left(Y0|D=1\right), \end{array}$$

where ATT is the average treatment effect on treated. In social experiments where treatment assignment is random, this is ensured, and the treatment effect is identified. *D*_*i*_ is whether household *i* has got the treatment or not (i.e., whether a household diversified or not).

The validity of the outputs of the PSM method depends on the satisfaction of two basic assumptions, namely, the conditional independence assumption (CIA) and the common support condition (CSC) [32]. CIA (also known as confoundedness assumption) states that given a set of observable covariates (*X*), which are not affected by treatment (in our case, diversification), four commonly used matching algorithms, namely, nearest-neighbor matching (NNM), caliper matching (CM), kernel-based matching (KM), and radius matching (RM), were employed to assess the impact of livelihood diversification on households' poverty. The NNM method matches each household from the diversified group with the non-diversified group having the closest propensity score. The matching can be done with or without the replacement of observations. NNM faces the risk of bad matches if the closest neighbor is far away. The caliper matching (CM) method uses a weighted average of all households in the diversified group to construct a counterfactual. The bandwidth is similar between kernel-based matching (KM) and radius matching (RM). The appropriate matching algorithm should be selected by observing three criteria in the result that are: the balancing test, the reduction in standard, pseudo-*R*^2^, and matched sample size [33]. Thus, a matching algorithm that balances the most explanatory variables, results in a low pseudo-*R*^2^ value, reduces more standard bias, and also results in a large matched sample size should be selected.

### 2.6. Variable Definition and Working Hypothesis

Dependent variable: household livelihood diversification is a dichotomous variable representing household diversification, taking a value of 1 if the household is diversified, and 0 if not. The livelihood diversification situation of a household is identified by the main livelihood activities pursued by a household. Households that generate their income only from agriculture were considered as non-diversified, while households that derive additional income from non- or off-farm activities were considered to be participating in livelihood diversification. However, the outcome variable to assess the impact of livelihood diversification on household poverty is those households participating in any livelihood diversification activities that earn additional income from off-farm sources, representing households that are not poor or otherwise poor. The independent variables were specified in Table 1.

## 3. Results and Discussion

### 3.1. The Sample Household's Demographic and Socioeconomic Characteristics

The survey result indicates that the study sample respondents were dominated by male heads (77.66%), while only a few (22.34%) were female heads. The majority of the respondents (84.94%) were married. Relatively, the more mean-aged (43.25 years) respondents were those who diversified their activities into different livelihoods. The mean of sample household heads' education level was 3.51 years of schooling with diversified sources. The result indicates that the mean sample household size of the respondents who were diversified in their major livelihood sources was 5.61. This result implies that sample households who have used a diversified source of livelihood have relatively more size than those who have not diversified. Moreover, of the total household members, the number of dependent family members found between age 15 and 64 years of economic activity was taken as an important variable for livelihood diversification. The livestock holdings in TLU for the respondents who were not diversified were about 2.93 TLU, whereas for those who were diversified, their livelihood was about 1.86 TLU. In the study area, the maximum and minimum land sizes of sample households are 3.75 and 0.125 hectares, respectively. However, in the study area, households were involved in a diversity of off- and non-farm income sources (61.04%), as compared to only farm alone (38.96%) of sample households.

### 3.2. Sample Households' Consumption Expenditure and Poverty Status

The quantitative measure of poverty was estimated by using food and non-food expenditure to set the poverty line. The food poverty line was calculated by selecting a basket of foods commonly consumed by households in the study area, such as maize, sorghum, teff, pulses, inset (kocho) potato, sweet potato vegetables, fruits, oil, milk, meat, and other stimuli such as coffee, tea, and chocolate. The quantity bundle of these meets the predetermined level of minimum calorie requirement and is valued at local prices on average to get a constant poverty line.

The distribution of households by estimated annual consumption expenditure per AE was computed from the survey data. For the entire sample of households, annual consumption expenditure per AE ranged from 1081 to 4135 Birr, with a mean of 2920 Birr. The overall actual household consumption expenditure per AE in the study area during survey 2019 clearly shows that the minimum subsistence requirement for most households was met. The sharing of household consumption expenditure per AE compared to the minimum amount required indicated that 2887 Birr is required per adult per year in order to ensure survival. This result is in line with the finding of [34] estimating the average time needed to exit poverty and identifying the determinants of rural household poverty.

**Figure 2:**
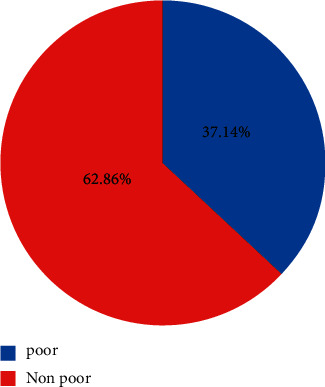
Poverty status of sample household.

This implies that households whose minimum amount required fell below the required per adult per year in order to be classified as poor, while households whose minimum amount required equaled or was above the poverty line to ensure survival was classified as non-poor (Figure 2). The figures illustrate that among the sample households presented using the approaches specified and discussed in the methodology part, 62.86% were non-poor (able to meet the minimum subsistence requirement), and the remaining 37.14% were poor.

#### 3.2.1. Incidence of Poverty in Sample Households

The results presented in (Table 2) show the values for the poverty measures, poverty headcount, poverty gap, and severity of poverty in the study area. The poverty measure (*P*_0_) developed by Foster et al. [29] was used to explain the extent of poverty in the study area. The resulting poverty estimates for the study area show that the percentage of poor people measured in the headcount index is low. The poverty headcount index (incidence of poverty) was computed for the study area and shows that poverty headcount (*H*)^0^, poverty gap (PG)^1^, and squared poverty gap (PG)^2^ were 0.3714, 0.026, and 0.086, respectively. The poverty gap index *P*(1), a measure that captures the mean aggregate consumption shortfall relative to the poverty line across the household, was 0.026, which means that the percentage of total consumption needed to bring the entire household to the poverty line is 2.6%. The results are in line with the findings of Adepoju and Obayelu [35] on livelihood diversification and the welfare of rural households below the poverty line and [36] poverty measures: 25 years later.

### 3.3. The Current State of Livelihood Diversification in the Study Area

**Figure 3:**
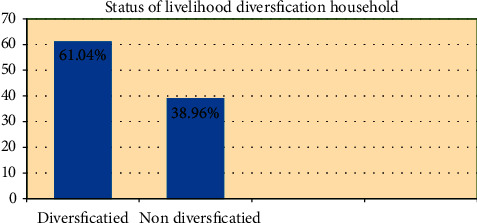
Status of livelihood diversification from the sample household.

Livelihood strategies may focus on increasing the range of assets to which a person or household has access or on increasing access to particular types of capital. The result (Figure 3) below indicated that in the study area, the majority 61.04% of the households were able to diversify their livelihoods into either of the three livelihood diversification strategies or combined income activities, whereas 38.96% of the sample households were unable to diversify their livelihoods, often lacking the means to engage in any form of income-generating activity.

Agricultural production and productivity are being hampered by the ongoing drought, limited farm, and grazing land, poor use of agricultural improved inputs, and other factors such as a lack of basic amenities, motivation, and interest in agricultural activities. The negative impact on food security and poverty on households may also severely affect the predicted rapid population growth in the future. As a result of this and other factors, the agricultural sector could not absorb the rural productive labor force. In reverse, it aggravates the already unbalanced farm livelihood situation of the study. According to Kassie and Aye [37], the declining size of farmland coupled with high population growth could have a potentially negative impact on rural welfare and food security in sub-Saharan Africa.

### 3.4. Econometric Results of the Determinants of Household Livelihood Diversification

Out of twelve explanatory variables included in the logistic model, six variables have shown statistical significance in determining rural households' livelihood diversification, while the remaining six do not show a significant relationship with rural poverty. Of the six significant variables, family size and credit services are highly significant at a 1% probability level. The variables of education level and livestock ownership are strongly significant at the 5% probability level, whereas off-farm income and landholding are at a 10% probability level (Table 3).

### 3.5. The Impact of Livelihood Diversification on Household Poverty

To identify the impact of livelihood diversification on the income of rural households, increasing probability, the study identified livelihood activities employed by households and poverty status of households. Moreover, the income share method was used to identify diversified and non-diversified households. The descriptive statistics for diversified and non-diversified households show that the two groups had a significant mean difference concerning the asset holdings of households. That means a logit model is used to identify the determinants of participation in different livelihood activities in rural poverty. To identify the impact of rural household poverty, the proportion of the population whose standard of living is greater than the poverty line to the number of individuals or households.

#### 3.5.1. The Nexus between Livelihood Diversification and Household Poverty in the Study Area

The survey result revealed that among 385 total sample households, the overall average number of households participating in different livelihood activities was 61.04%, and the remaining 38.96% were not participating in any income-earning activities. In addition, of the total sample households, 62.86% of the households are found to be non-poor (lying above the poverty line), while 37.14% of them are found to be poor (lying below the poverty line). The chi-square result in Table 4 indicates that 2.7840 Pr = 0.095. This result shows that the difference between livelihood diversification and poverty status is significant at a 10% probability level. This implies that most of the rural households in the study area diversify their livelihood sources, and this may influence their level of fairly strong linkage between poverty and diversification.

#### 3.5.2. Matching Group and Non-Group Households

The propensity score is computed based on the logistic model, and they serve as a tool to balance the observed distribution of covariates across the treated and the untreated group It was done using the “*p* score” command in STATA to predict a propensity score between the two groups. The estimation (Table 5) indicated that propensity scores vary between 0.150 and 0.978 (mean = 0.68) for diversified households and between 0.099 and 0.908 (mean = 0.496) for non-diversified (control) households. The common support region would therefore lie between 0.150 and 0.908, which means households whose estimated propensity scores are less than 0.150 and larger than 0.908 are not considered for the matching purpose. As a result of this restriction, 28 households were discarded.

#### 3.5.3. Algorithm Selection for Matching

A matching estimator that balances all explanatory variables with the lowest pseudo-*R*^2^ value and produces a large matched sample size is preferable to present the estimated results of tests of matching quality based on the three performance criteria. Looking into the result of the matching quality, nearest-neighbor (NN) matching of neighborhood 2 was found to be the best for the data we have at hand. Hence, the estimation of the results and discussion for this study are the direct outcomes of the NN matching algorithm with a neighborhood 2.

#### 3.5.4. Test for Propensity Score and Covariate Balances

The balancing powers of the estimations are ensured by different testing methods. Reduction in the mean standardized bias between the matched and unmatched households; equality of means using t-test and chi-square tests for joint significance of the variables used is employed here. The standardized difference in covariates before matching is in the range of 3.7% to 65.2% in absolute value, whereas the remaining standardized difference in covariates for almost all covariates lies between 1.1% and 13% after matching. This is fairly below the critical level of 20% suggested by [33].

As indicated in (Table 6), the values of pseudo-*R*^2^ are very low (0.007). This low pseudo-*R*^2^ value and the insignificant likelihood ratio tests (*P* > chi^2^ 0.993) support the hypothesis that both groups have the same distribution in the covariates after matching. These results indicate that the matching procedure is able to balance the characteristics of the treated and the matched comparison groups. Hence, these results can be used to assess the impact of diversification among groups of households having similar observed characteristics.

#### 3.5.5. The Common Support Condition

**Figure 4:**
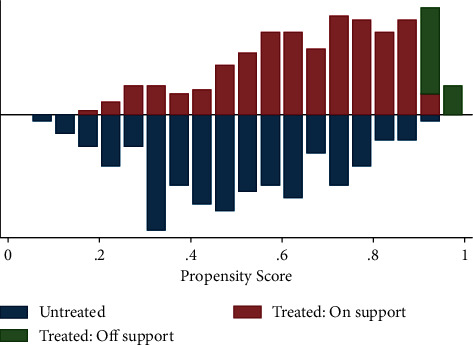
Propensity score matching graph.

Figure 4 gives the histogram of the estimated propensity scores for diversified and non-diversified. A visual inspection of the density distributions of the estimated propensity scores for the two groups indicates that the common support condition is satisfied: there is substantial overlap in the distribution of the propensity scores of both groups. The bottom half of the graph shows the propensity score distribution for the non-diversified/untreated, and the upper half refers to the diversified/treated on support. The densities of the scores are on the *y*-axis [38].

#### 3.5.6. The Effect of Treatment on the Treated

In order to attain the stated objectives of the study, this section evaluates the impact of the diversification on the outcome variables for their significant impact on participant households, after the preintervention differences were controlled. The estimation result presented in Table 7 provides supportive evidence of the significant effect of livelihood diversification on the outcome variable. A positive value of average treatment is its effect on the treated (the difference between the treated and the control) due to the participating household livelihood diversification decreasing the poverty status.

The propensity score matching results showed that participation in different livelihood diversification activities has a significant effect on the households' consumption expenditure level over the poverty line. The average treatment effect of treated indicated that the average annual per capita consumption expenditure of participants is more than that of the non-participated households. Thus, participation in livelihood diversification activities improved the income of households in the study area.

The estimation result provides supportive evidence of the significant and positive effect of the household poverty status in Birr by consumption of expenditures on food and non-food households, which means the cost of basic needs. Participation in livelihood diversification has increased the income of the households in Birr for participant households on average by 9%. This finding is in line with [39] who found the positive impacts of livelihood diversification on households' food security, [40] who found the positive impact of livelihood diversification on household income, and [41] who found the positive impact of non-farm activities on poverty reduction among rural farm households.

## 4. Conclusion and Recommendations

Agriculture is the dominant economic activity and the primary source of livelihood in rural households. However, due to small farm size and uncontrolled population growth, agricultural production has declined over time and has forced people to look for alternative employment options other than agriculture for achieving food security and reducing poverty in the rural area. Furthermore, the survey results also reveal the fact that rural households in the study area practice diversified livelihood strategies rather than agriculture.

The study conduct cost of basic needs approaches computed the poverty line of the household study area by using consumption as an indicator of the FGT poverty line measure of welfare or standard of living. Based on the information on the welfare indicator of adult equivalent consumption, we computed the poverty line, which is the combination of food and non-food poverty expenditure, 2887 Birr. Considering the amount of benchmark, the poverty line result shows that the proportion of households below the poverty line was 37.14% poor (under requirement) and 62.86% of the sample households were non-poor (able to fulfill the basic requirement).

The result of the logit model indicated that factors family size, landholding, livestock ownership, year of schooling, access to credit services, and off-farm income of the households were found to have significantly determined livelihood diversification. Moreover, the results of the propensity score matching indicate that household participation in livelihood diversification has a positive and significant impact on household poverty. On the other hand, the average of participating in different livelihood activities was 61.04%, and the remaining 38.96% were not participating in any income-earning activities. This implies that most of the rural households in the study area diversify their livelihood sources, and this may influence their level of fairly strong linkage between poverty.

The impact estimate result shows that the participants' livelihoods had positively influenced household poverty in the study area. Participation in livelihood diversification has increased the income of participant households on average by 9% of what they would have had in the non-diversified. The implication is that rural livelihood diversification plays a vital role in reducing poverty and increasing the incomes of rural households.

The study suggests that diversified economic activities provide rural households in the study areas with an opportunity to manage household food security, reduce poverty, and improve living conditions. Income from off- or non-farm sector is likely to enable rural households to increase their purchasing power, enabling increased expenditure on food and consequently increasing access to income. Generally, government and policymakers should recognize and support off- and non-farm livelihood diversification strategies as part of the study area's job creation objectives instead of increasing rural income and reducing rural poverty, which strongly relies upon the development of off- or non-farm activities.

## Figures and Tables

**Table 1:** Variables and work hypothesis summary table.

Dependent variables		Dummy variables (1 = if household livelihood activities are diversified; 0 = if non-diversified)	
Outcome variable		Dummy (HH poverty status 1 = if poor, 0 = if not poor)	
Independent variables			
Variable	Variable type	Variable definition/measurement	Expected sign
AGE HHD	Continuous	Age of household head measured in years	+ve
SEX HHD	Dummy	Sex of household head representing 1, male; 0, female	+ve
FAM SIZE	Continuous	Number of family members in a household	+ve
DEPENDRT	Continuous	The ratio of (children under the age of 15 and old age of above 65 to the active labor force)	−ve
EDU	Continuous	Education level of household head in the year of schooling	+ve
LAND	Continuous	Land size owned in hectares	−ve
TLU	Continuous	Total livestock ownership in tropical livestock unit	−ve
DISMAK	Continuous	Distance from house to nearest local market measured by kilometer	+ve
CREDIT	Dummy	Access to credit service, 1 if the household received; 0 otherwise	+ve
EXTENSION	Continuous	Frequency of extension contact of days within a month	+ve
OFFF INC	Continuous	Annual income get from off-farm activities in ETB	+ve
NON INC	Continuous	Annual income get from non-farm activities in ETB	+ve

Field survey (2019).

**Table 2:** Poverty measures for households in the study area (*N* = 385).

	Poverty measure	Index value
	Poverty line	2887.1/year/AE
(*H*)^0^	Poverty head count (*H*)^0^	0.3714
(PG)^1^	Poverty gap	0.026
(PG)^2^	Squared poverty gap	0.086

Authors' calculated own survey data (2019).

**Table 3:** Robust standard error of logit model before impact estimation.

Variables	Coefficient (*β*)	Robust std. err	*z*	*P* > *z*
Sex	0.2398855	0.244918	0.98	0.327
Age	0.0022859	0.0090128	0.25	0.800
Family size	0.3254973	0.0617953	5.27^*∗∗∗*^	0.001
Dependency ration	−0.0991398	0.1533176	−0.65	0.518
Education	0.097415	0.0464259	2.10^*∗∗*^	0.036
Credit	0.9661014	0.255318	3.78^*∗∗∗*^	0.001
Land size	−0.1440323	0.0827905	−1.74^*∗*^	0.082
Extension contact	0.1218438	0.1048981	1.16	0.245
Market distance	0.0012969	0.0031306	0.41	0.679
Livestock ownership	−0.1426449	0.0672762	−2.12^*∗∗*^	0.034
Off-farm income	0.000307	0.0001652	1.86^*∗*^	0.063
Non-farm income	0.0000661	0.0000456	1.45	0.147
Cons	−2.48189	0.8581793	−2.89	0.004

Number of obs = 385, Wald chi^2^ (12) = 63.20, pseudo-*R*^2^ = 0.1528, Prob > chi^2^ = 0.001, and log pseudo-likelihood = −218.07066. ∗∗∗, ∗∗, and ∗ represent significant at the 1%, 5%, and 10% probability levels, respectively. Computed own survey data (2019).

**Table 4:** Livelihood strategies by poverty status.

Livelihood diversification				
Poverty status	Diversified (*N* = 235)	Non-diversified (*N* = 150)	Total (*N* = 385)	*χ* ^2^
	%	%	%	
Non-poor (242)	39.48	23.38	62.86	
Poor (143)	21.56	15.58	37.14	
Total	61.04	38.96	100	2.784^*∗*^

∗ represents significant at 10% probability level. Computed own survey data (2019).

**Table 5:** Distribution of estimated propensity scores.

Group	Observation	Mean	SD	Minimum	Maximum
Total households	385	0.61	0.21	0.099	0.978
Diversified HHs	235	0.68	0.19	0.150	0.978
Non-diversify HHs	150	0.496	0.193	0.099	0.908

Computed own survey data (2019).

**Table 6:** Chi-square test for the joint significance of variables.

Sample	Ps *R*^2^	LR chi^2^	*P* > chi^2^
Unmatched	0.154	79.49	0.001
Matched	0.007	3.83	0.993

Computed own survey data (2019).

**Table 7:** Average treatment effect on the treated (ATT) estimation results.

Variable	Sample	Treated	Controls	Difference	SE	*t*-test
Change in poverty status in birr	Unmatched	2911	2593	317	75	4.22
	ATT	2913	2651	261	120	2.17^*∗∗*^

∗∗ represents significant at 5% probability level. Computed own survey data (2019).

## Data Availability

The data used to support the findings of this study are available from the corresponding author upon request.
